# Maturation trajectories and transcriptional landscape of plasmablasts and autoreactive B cells in COVID-19

**DOI:** 10.1016/j.isci.2021.103325

**Published:** 2021-10-23

**Authors:** Christoph Schultheiß, Lisa Paschold, Edith Willscher, Donjete Simnica, Anna Wöstemeier, Franziska Muscate, Maxi Wass, Stephan Eisenmann, Jochen Dutzmann, Gernot Keyßer, Nicola Gagliani, Mascha Binder

**Affiliations:** 1 Department of Internal Medicine IV, Oncology/Hematology, Martin-Luther-University Halle-Wittenberg, Ernst-Grube-Straße 40, 06120 Halle (Saale), Germany; 2 I. Department of Medicine and Department for General, Visceral and Thoracic Surgery, University Medical Center Hamburg-Eppendorf, Hamburg, Germany; 3 Department of Internal Medicine I, Martin-Luther-University Halle-Wittenberg, 06120 Halle (Saale), Germany; 4 Mid-German Heart Center, Department of Cardiology and Intensive Care Medicine, University Hospital, Martin Luther University Halle-Wittenberg, 06120 Halle (Saale), Germany; 5 Department of Internal Medicine II, Martin-Luther-University Halle-Wittenberg, 06120 Halle (Saale), Germany; 6 Hamburg Center for Translational Immunology (HCTI), University Medical Center Hamburg-Eppendorf, Hamburg, Germany; 7 Immunology and Allergy Unit, Department of Medicine, Solna, Karolinska Institute and University Hospital, Stockholm, Sweden

**Keywords:** Immunology, Virology, Transcriptomics

## Abstract

In parasite and viral infections, aberrant B cell responses can suppress germinal center reactions thereby blunting long-lived memory and may provoke immunopathology including autoimmunity. Using COVID-19 as model, we set out to identify serological, cellular, and transcriptomic imprints of pathological responses linked to autoreactive B cells at single-cell resolution. We show that excessive plasmablast expansions are prognostically adverse and correlate with autoantibody production but do not hinder the formation of neutralizing antibodies. Although plasmablasts followed interleukin-4 (IL-4) and BAFF-driven developmental trajectories, were polyclonal, and not enriched in autoreactive B cells, we identified two memory populations (CD80^+^/ISG15^+^ and CD11c^+^/SOX5^+^/T-bet^+/−^) with immunogenetic and transcriptional signs of autoreactivity that may be the cellular source of autoantibodies in COVID-19 and that may persist beyond recovery. Immunomodulatory interventions discouraging such adverse responses may be useful in selected patients to shift the balance from autoreactivity toward long-term memory.

## Highlights

• Plasmablast expansions correlate with disease severity and autoantibodies in COVID-19
• Patients with high plasmablast levels exhibit IGHV4-34 skewing
• Autoreactive BCRs are enriched in atypical memory, not plasmablast populations

## Graphical abstract

**Figure:**
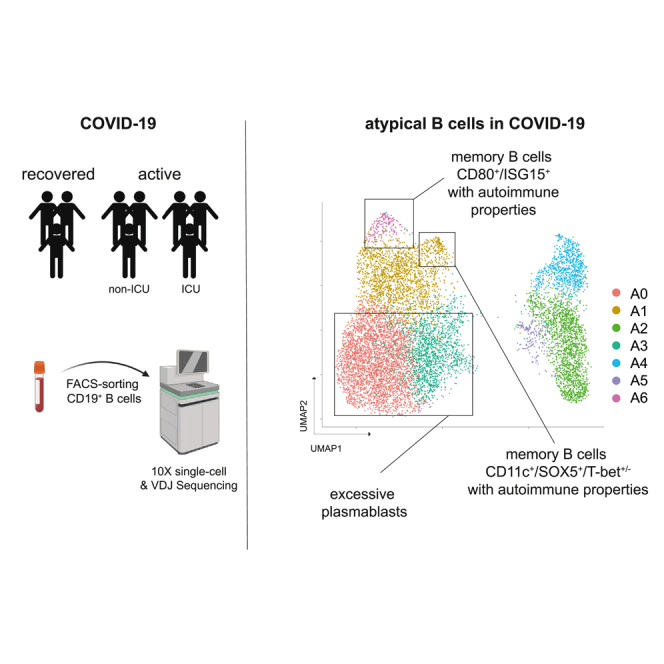

## Introduction

Antibody generation is an essential part of the adaptive immune response and the basis for neutralization of infectious agents as well as vaccination success. Antigenic encounter triggers B cell proliferation, resulting in generation of a small subset of antibody-secreting short-lived plasmablasts (PBs). This extrafollicular response that may also generate atypical memory B cells occurs outside germinal centers (GC) and rapidly generates antibodies (MacLennan et al., 2003). In the second wave of the B cell response, some of the activated, cycling B cells engage in a GC reaction, the B cell receptor (BCR) undergoes affinity maturation through somatic hypermutation (SHM) (Laidlaw and Cyster, 2020), and some of the cells develop into memory B cells or long-lived plasma cells. In some infectious diseases prolonged “noncanonical” extrafollicular responses occur that seem to induce immunopathology and may have a negative impact on GC reactions and long-lived memory formation. The factors and mechanisms driving these pathological reactions are, however, far from being settled (Elsner and Shlomchik, 2020; MacLennan et al., 2003). One such example is the elevated PB responses subverting humoral immunity in malaria by outcompeting GC reactions through metabolic constraints (Vijay et al., 2020). In an animal model, therapeutic administration of a single amino acid to experimentally infected mice was sufficient to overcome the metabolic constraints imposed by PBs, thereby enhancing parasite clearance and immunological memory. In dengue virus infection, due to the co-occurrence of substantial PB reactions and disease deterioration it has been hypothesized that this response may relate to the immunopathology observed in this viral infection (Wrammert et al., 2012). In addition, circulating PBs have been found to be responsible for the generation of disease-specific largely unmutated autoantibodies generated during flares of systemic lupus erythematosus (SLE) (Tipton et al., 2015). This is especially interesting because in malaria, where excessive PB responses seem to impair GC reactions, autoantibodies (some of which with clear clinical implications such as autoimmune hemolysis) can be induced, thereby creating a pathophysiological link between extrafollicular responses and autoimmunity (Jenks et al., 2019; Rivera-Correa et al., 2017).

In COVID-19, large PB expansions were noted as a characteristic feature very early on in the pandemic (Bernardes et al., 2020; De Biasi et al., 2020; Kuri-Cervantes et al., 2020; Mathew et al., 2020; Wildner et al., 2021). Further characterization showed that these reactions resulted in high SARS-CoV-2 antibody titers, yet being closely linked to fatal clinical courses (Woodruff et al., 2020). This was supported by independent investigations showing a lack of GC formation in the lymph nodes of patients who died as a consequence of COVID-19 (Kaneko et al., 2020). Interestingly, parallels of this extrafollicular response to B cell repertoire features previously described in autoimmune settings such as SLE were noted (Woodruff et al., 2020).

Here we link B cell receptor sequences with transcriptional B cell programs in COVID-19 in order to determine the so far unrecognized cellular source of autoantibodies in this disease. Although expanded PBs were clearly linked to plasma autoantibodies in our patients, we found evidence that not the PBs themselves, but atypical B cells with low T-bet expression as well as CD80^+^/ISG15^+^ memory B cells harbor immunogenetic and transcriptional signs of autoreactivity in COVID-19.

## Results

### Prognostically unfavorable expansion of CD20^dim/−^ plasmablasts in individuals with acute COVID-19

**Figure 1:**
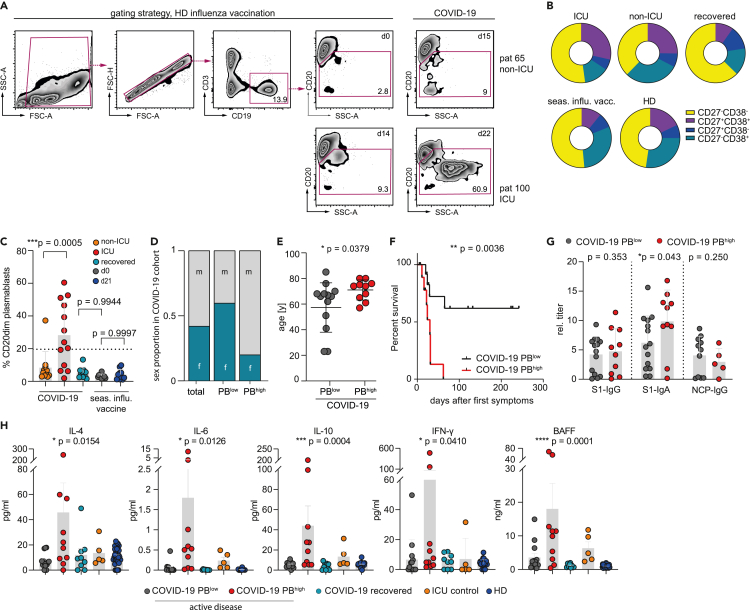
Flow cytometric plasmablast identification and profiling of soluble factors in COVID-19 patients (A) Exemplified flow cytometric (FC) gating strategy to identify CD20^dim/−^ plasmablasts (PBs) from the CD19^+^CD3^−^ B cell pools of 23 COVID-19 patients within the first four weeks of infection. Ten recovered patients and ten individuals vaccinated with the seasonal flu vaccine (VaxxigripTetra2020/2021) served as control. (B) Proportion of CD27^−^/CD38^−^, CD27^−^/CD38^+^, CD27^+^/CD38^+^, and CD27^+^/CD38^−^ cells in the CD20^dim/−^ PB populations from (A). (C) Summary of CD20^dim/−^ PB expansions as detected in 23 COVID-19 patients by FC. Individuals with PB expansions ≥20% of the CD20 pool are considered as PB^high^ (n = 10). Error bars indicate mean ± SD. Statistics: ordinary one-way ANOVA followed by post-hoc testing (Tukey's multiple comparisons test). (D and E) Correlation of PB^high^ phenotype with sex (D) and age (E). Error bars indicate mean ± SD. Statistics: two-sided t test. (F) Kaplan-Meier survival analysis of PB^high^ (n = 10) versus PB^low^ (n = 13). Statistics: logrank test. (G) Plasma titer of SARS-CoV-2 antibody classes directed against the S1 and NCP protein in PB^high^ (n = 10) and PB^low^ (n = 13) COVID-19 patients. Error bars indicate mean ± SD. Statistics: two-sided t test. (H) Mean plasma levels of key cytokines for B cell function in PB^high^ (n = 10) and PB^low^ (n = 13) COVID-19 patients and follow-up samples at recovery (n = 8) compared with patients with bacterial pneumonia and HDs (n = 32). All samples were measured at least in duplicates. Error bars indicate mean ± SEM. Statistical analysis: ordinary one-way ANOVA. ICU, pneumonia patients requiring intensive care; HD, healthy donor. ∗p < 0.05; ∗∗p < 0.01; ∗∗∗p < 0.001; ∗∗∗∗p < 0.0001.

We quantified peripheral PBs by flow cytometry within 4 weeks after disease onset (median sampling at day 15, range 0–26; Figures S1A and S1B) in active COVID-19 patients (pt) as well as d14 ± 7 after influenza vaccination using a broad gating strategy (CD3^−^/CD19^+/dim^/CD20^dim/−^/SSC^dim or high^) to cover all stages of PB development as previously described (Jourdan et al., 2011; Sanz et al., 2019) (Figures 1A–1C). The active COVID-19 cohort showed substantial expansions of CD20^dim/−^ PBs averaging 19.15% (range 1.87%–60.4%) of circulating CD19^+^ B cells, whereas increased levels of CD20^dim/−^ PBs were not observed 14 (+/−7) days after influenza vaccination (mean 4.19%, range 1.12%–9.81%) or in healthy individuals (3.3%, range 1.73%–6.05%) (Figures 1A and 1C). We also observed a trend of persisting CD20^dim/−^ PB expansions in recovered PCR-negative individuals 4–7 weeks after disease onset (median sampling at day 39, range 30–49), although at low levels (Figure 1B). The CD20^dim/−^ PB populations of COVID-19 patients also encompassed more CD27^+^CD38^+^ cells as compared with recovered patients or healthy individuals (HD) (Figure 1B). PBs constituted more than 20% of all B lineage cells (PB^high^) in 10 of 23 patients. These patients had a substantially higher requirement for ICU treatment (Figure 1C), were predominantly male (Figure 1D), and were of older age (Figure 1E). The severe disease course of PB^high^ COVID-19 patients was also reflected by their high mortality (8 out of 10) (Figure 1F), which is in line with previous observations (Woodruff et al., 2020). Although less clear, potentially due to sample size, we observed a similar correlative trend with disease severity when focusing on CD19^+^CD27^+^CD38^+^ PBs (Figures S1C–S1E). Immunosequencing revealed that the BCR repertoires of PB^high^ COVID-19 patients were less diverse, more clonal, and associated with higher levels of SHM as compared with PB^low^ patients, recovered individuals, and healthy controls (Figure S1F). In addition, we did not detect any differences in complementarity-determining region 3 (CDR3) length distribution or amino acid composition between groups (Figures S1G–S1I).

### COVID-19 patients with high CD20^dim/−^ plasmablast counts show normal SARS-CoV-2 antibody titers but a specific cytokine pattern

S1- or nucleocapsid protein (NCP)-specific SARS-CoV-2 IgG antibody titers did not appear to differ between patients with high CD20^dim/−^ plasmablast counts and the rest of the cohort, whereas S1-IgA titers were more abundant in the PB^high^ group (Figure 1G). Moreover, patients with high CD20^dim/−^ plasmablast levels showed a cytokine profile including high levels of interferon gamma (IFN-γ) and interleukin-6 (IL-6) consistent with the severity of their disease (Figure 1H). These patients also had high plasma levels of BAFF, IL-10, and IL-4, which are known to promote the generation and survival of plasmablasts (Figure 1H). Consistent with prior evidence, antibody titers decreased over time in almost all patients with adequate follow-up (Figure S1J).

### Patients with high CD20^dim/−^ plasmablast counts show a high likelihood for autoantibody formation in COVID-19

**Figure 2:**
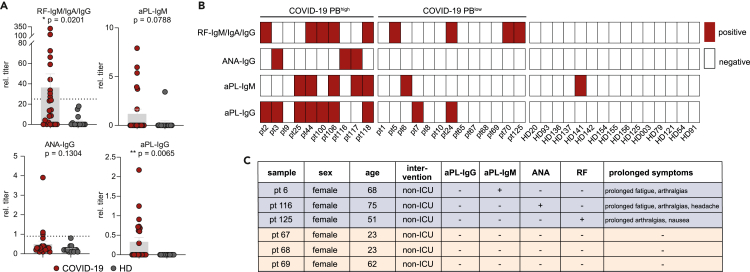
Autoantibodies in COVID-19 patients (A) Detection of rheumatoid factor (RF), antinuclear (ANA), and phospholipid autoantibodies (aPL) in the COVID-19 patients (pt; n = 23) as compared with HDs (n = 15). Depicted as mean relative antibody titer ± SEM. Statistics: two-sided t test Welch corrected. ∗p < 0.05; ∗∗p < 0.01. (B) Patient-specific seropositivity of autoantibodies in the PB^high^ (n = 10) and PB^low^ (n = 13) COVID-19 groups as compared with HD (n = 15). (C) Persisting COVID-19 symptoms (long-COVID) reported from patients during follow-up examinations.

The association of excessive plasmablast expansions with high BAFF, IL-10, and IL-4 levels led us to speculate that the plasmablasts could be partially unspecific and may contain B cell receptor sequences not reactive with SARS-CoV-2 epitopes. Because prior work already pointed at parallels between extrafollicular responses in COVID-19 and systemic lupus erythematosus (SLE) (Woodruff et al., 2020) and given that autoantibodies have been reported in the context of COVID-19 (Borghi et al., 2020; El Hasbani et al., 2020; Vlachoyiannopoulos et al., 2020; Zuo et al., 2020), we reasoned that the plasmablasts may—at least partially—contain autoreactive B cell receptors and produce autoantibodies. Screening for rheumatoid factor (RF), antinuclear antibodies (ANA), and phospholipid antibodies (aPL) in our COVID-19 cohort revealed elevated levels of all classes (Figure 2A). Notably, autoantibodies were enriched in the PB^high^ subset (Chi square p = 0.04) with 9 out of 10 PB^high^ individuals exhibiting seropositivity for at least one of the measured classes, whereas in the remaining cases only 6 out of 14 were autoantibody positive (Figure 2B). Despite the well-established sexual dimorphism observed in autoimmune diseases (Rubtsova et al., 2015), there was no female predominance in the autoantibody-positive COVID-19 patient subset (Figure S2). In a small subset of six female patients, symptom monitoring could be performed over 12 weeks after infection. Of note, persistent symptoms such as fatigue, exhaustion, and muscle and joint pain—commonly referred to as “long-COVID”—were recorded only in autoantibody positive patients (Figure 2C).

### COVID-19 B lineage single-cell RNA sequencing reveals transcriptome of expanded CD20^dim/−^ plasmablast population

**Figure 3:**
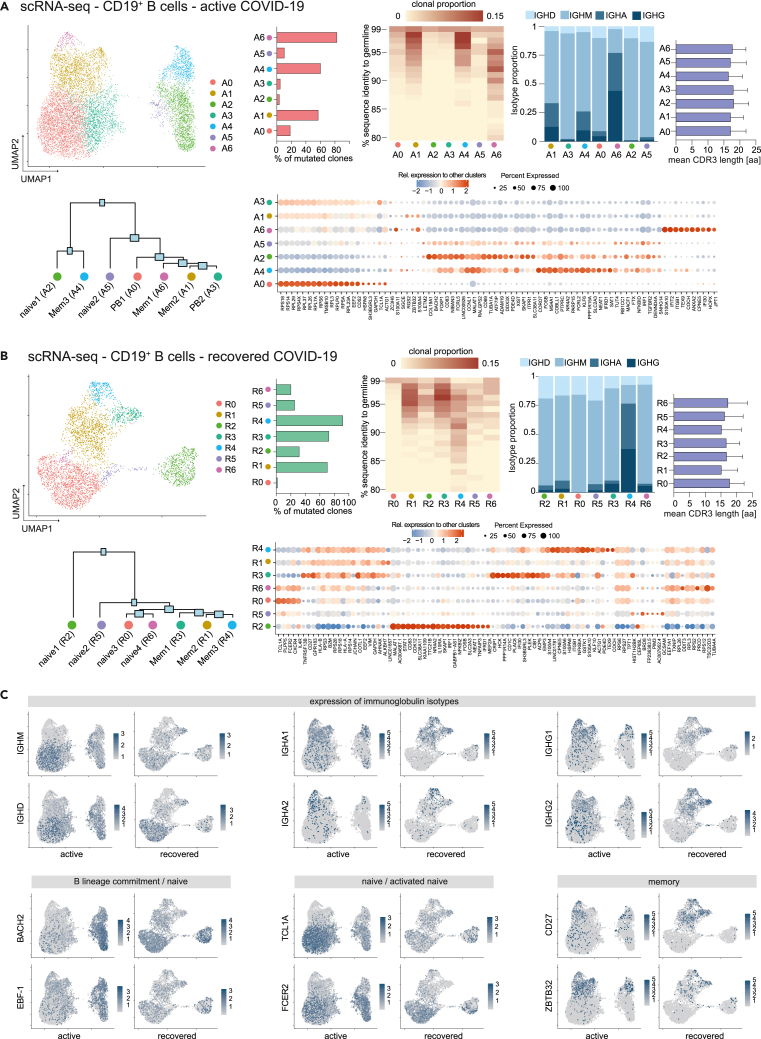
Single-cell RNA and V(D)J sequencing of COVID-19 CD19^+^ B cells (A–C) B cells from seven COVID-19 patients (4 active, 3 recovered) were sorted by flow cytometry and subjected to single-cell RNA sequencing. Uniform Manifold Approximation and Projection (UMAP) plot displaying the 7 cell clusters identified in the active group (A0-6, 10,050 cells) (A) and the recovered group (R0-1, 4,119 cells) (B). Unsupervised hierarchical clustering of gene signatures indicates similarity relations between the COVID-19 clusters. The mean rate of somatic hypermutation (SHM), the isotope proportion, and mean CDR3 length (error bars indicate mean ± SD) for each cluster are depicted as (stacked) bar plots; the top 20 differentially expressed genes for each are shown as dot plots. The clonal proportion across the hypermutation spectrum is depicted as heatmap. (C) UMAP showing expression of genes associated with key features of B cell subset development and function.

**Figure 4:**
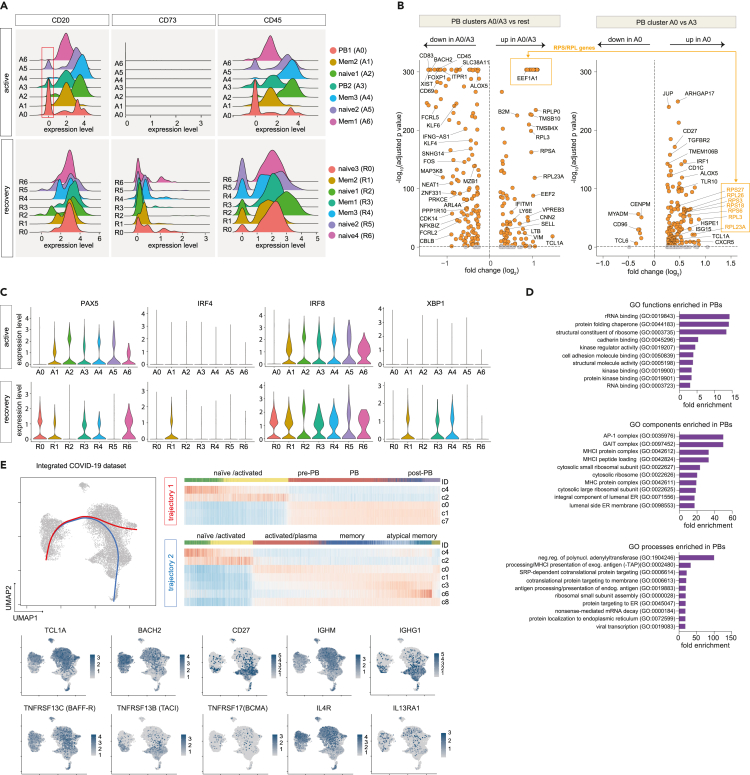
Definition of plasmablast populations by single-cell RNA sequencing (A) Density plots indicating plasmablast (PB) subsets based on reduced CD20, CD73, and CD45 expression levels (red box). (B) Differentially expressed genes between PB populations (A0/A3) and remaining clusters as wells as between populations A0 and A3. Genes with adjusted p < 0.01 and log_2_ fold change < or >0.5 were labeled orange. (C) Distribution of cells expressing plasma cell differentiation markers across clusters. (D) The top 10 enriched biological functions, processes, and components (GO terms) in the A0 population. (E) UMAP of all integrated COVID-19 samples and expression signatures of key genes for B cell categorization. Developmental trajectories computed using the slingshot package were plotted on the UMAP; the corresponding expression patterns are shown as heatmap.

To dissect the molecular profiles of peripheral B cells in COVID-19, we subjected seven COVID-19 patients (four with active infection: pt 2, 3, 5, 8; three after recovery: pt 14, 16, 26) (Table S1; Figure 2) as well as one HD to single-cell RNA and V(D)J sequencing. After correction for read depth and mitochondrial transcripts, 10,050 cells were obtained for the active COVID-19 group, 4,119 for the recovered, and 1,133 for the control individual. To assess the quality of our dataset, we integrated all cells into a recently published set of 12,973 peripheral CD19^+^ B cells from three healthy individuals (Stewart et al., 2021). Dimensionality reduction and visualization of transcriptomic profiles using graph-based clustering of uniform manifold approximation and projection (UMAP) yielded eleven distinct cell clusters with contributions from each individual compatible with the populations described by Stewart et al. (2021) (Figure S3). Next, we examined differences in the transcriptional profile of B cells during active disease and after recovery by integration of the respective COVID-19 samples (Figures 3A–3C). This analysis revealed the presence of seven clusters for each dataset (A0-6 and R0-6), which were manually assigned to conventional B cell subsets based on the expression of established markers (Glass et al., 2020; Horns et al., 2020; Stewart et al., 2021) (Figures 3A and 3B). Both cohorts showed several populations with a naive/activated B cell profile (*BACH2*, *EBF-1*, *TCL1A*, *FCER2*) and preswitch isotypes (A2, A5 and R0, R2, R5, R6) as well as predominantly isotype-switched B cells with a memory-like expression pattern (*CD27*, *ZBTB32*) and high rates of SHM (A1, A4, A6 and R1, R3, R4) (Figures 3A–3C). In line with the bulk immunosequencing data (Figures S1F–S1I), we did not observe substantial differences in CDR3 length between B cell subsets in both groups (Figures 3A and 3B). Combination of the top 100 differentially expressed genes for each cluster revealed involvement of B cells in interleukin signaling, especially IL-4, and IFN responses as well as deregulation of metabolic and proliferative processes (Figure S4). In line with the flow cytometry data (Figures 1A and 1B), clusters A0 and A3 were identified as PBs based on their reduced *MS4A1* (*CD20*), *CD73*, and *PTPRC* (*CD45*) expression (Figures 3A and 4A–4C). Both populations shared loss of *CD20* and upregulation of genes associated with metabolic processes and autophagy (ribosomal genes, *HSPA8*), which was more pronounced in A0 (Figures 3B, 4B and 4D). Both clusters were also characterized by downregulation of proliferation (*MAP3K8*, *FOSB*, *CDK14*), activation, and differentiation (*BACH2*, *CD69*, *CD83*) markers (Figure 4B). *PAX5* and *IRF8* downregulation in conjunction with upregulation of the BLIMP-1/PRDM1 surrogate *IRF1*(Kuo and Calame, 2004; Minnich et al., 2016; Scharer et al., 2018) in A0 suggests this cluster as terminally differentiated PBs and A3 as an early or pre-PB population (Figure 4C). This was corroborated by pseudotime analysis of all integrated COVID-19 B cells (Figure 4E). The inferred developmental trajectories suggest that although A0 PBs–most likely driven by IL4R- and TNFRSF13C (BAFF-R)-mediated signaling—directly emerge from naive B cell populations (trajectory 1), development of the memory B cell pool included an intermediate activated naive B cell population (Figure 4E). In addition, PBs also showed differential expression of IFN-response genes, including *IFITM1* (Yang et al., 2007), *ISG15* (Perng and Lenschow, 2018), *IFNG-AS1* (Rankin et al., 2020), *NEAT1* (Suarez et al., 2020), and *NKBIZ* (Ishiguro-Oonuma et al., 2015) (Figure 4B), and of the homing receptor *CD62L* (*SELL*) (Figure 4B). No similar PB clusters were found in the healthy, uninfected subject (Figures 3 and 4). Notably, the recovered individuals contained clustered cells with a classical signature of plasma cell activity (*XBP1*) that was not detected during active disease (Figure 4C).

### SARS-CoV-2-specific B cells predominantly show preswitch isotypes and distribute evenly across all major B cell subsets in COVID-19

**Figure 5:**
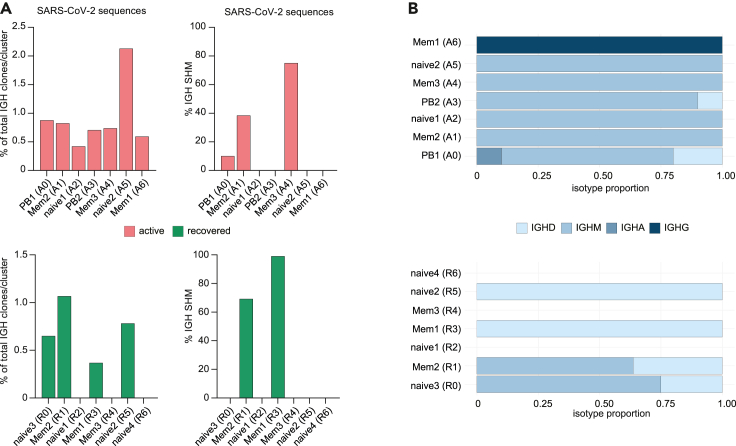
Distribution of SARS-CoV-2-specific B cell receptor sequences across B cell subsets (A) SARS-CoV-2 epitope-specific V(D)J rearranged B cell receptor sequences retrieved from CoVAbDab (Galson et al., 2020), IEDB (Vita et al., 2019), and (Ehling et al., 2021) were searched in the V(D)J single-cell data. Distribution of these B cell clones is shown across clusters, including proportion of SARS-CoV-2 epitope-specific clones with somatic hypermutation (SHM) per cluster. (B) Isotype of SARS-CoV-2 epitope-specific clones.

V(D)J rearrangements identified by single-cell sequencing of B cells from patients with COVID-19 were screened for CDR3 sequences known to interact with SARS-CoV-2 epitopes (Ehling et al., 2021; Galson et al., 2020; Vita et al., 2019). Forty-nine sequences were detected across all subpopulations in active disease and in 4/7 populations after recovery, with highest proportions in the naïve-like population A5 and the memory-like population R1 (Figure 5A). Consistent with previous findings (Galson et al., 2020; Kaneko et al., 2020; Kreer et al., 2020; Schultheiss et al., 2020; Seydoux et al., 2020; Woodruff et al., 2020), these BCR showed low levels of SHM and predominantly preswitch isotypes (Figures 5A and 5B). However, most SARS-CoV-2-specific BCRs in the memory populations A4, R1, and R3 showed imprints of affinity maturation (Figure 5A).

### B cells from COVID-19 patients show imprints of autoreactivity

**Figure 6:**
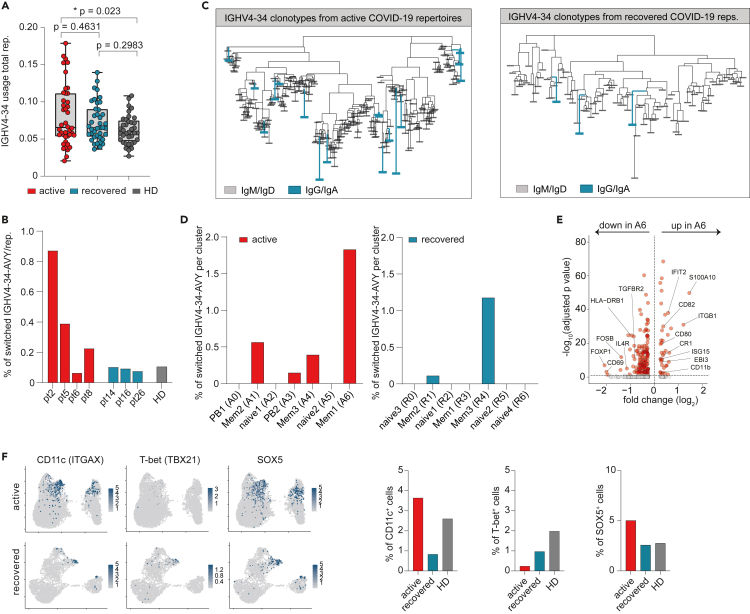
Features of CD19^+^ B cells from patients with COVID-19 related to autoimmunity (A) IGHV4-34 gene usage in active COVID-19 (n = 42), after recovery (n = 40), and HDs (n = 37) as detected by bulk IGH NGS. Plot indicates mean frequencies (with min to max range) per repertoire. Statistics: ordinary one-way ANOVA followed by post-hoc testing (Tukey's multiple comparisons test). Asterisks indicate p value range (∗p < 0.05). (B) Percentage of autoreactive isotype-switched IGHV4-34-AVY B cells in COVID-19 patients and HD. (C) Sequence clustering of IGHV4-34-AVY B cells in COVID-19 patients. Autoreactive sequences with post-switch isotypes are marked in teal. (D) Percentage of isotype-switched IGHV4-34-AVY B cells per subset. (E) Differentially expressed genes between A6 and all other cells from the active cohort. Genes with adjusted p < 0.01 and log_2_ fold change < or >0.5 were labeled orange. (F) UMAPs with expression of *CD11c* (*ITGAX*), *T-bet* (*TBX21*), and *SOX5* to identify atypical memory B cells. Percentage of cells positive for these markers within the complete active, recovered, and HD datasets are shown as bar plots. The HD dataset is comprised of the one individual from this study and the three published by (Stewart et al., 2021).

Because plasma autoantibodies were preferentially found in COVID-19 patients of the PB^high^ subset, we reasoned that they might directly be generated by the PB population. Because RF, ANA, and PL antibodies do not have defined sequences that could be searched in our dataset, we used IGHV4-34-expressing B cells with the framework region 1 AVY motif in its wild-type configuration (IGHV4-34-AVY) as proxy for autoreactive B cells. IGHV4-34-AVY encode intrinsically self-reactive antibodies and are therefore strongly discouraged to undergo class switch recombination in the healthy B cell repertoire (Bashford-Rogers et al., 2018; Li et al., 1996; Potter et al., 2002). By bulk NGS IGH immunosequencing of our patients including samples from our SARS-CoV-2 sequence repository (Schultheiss et al., 2020), we found a discrete expansion of IGHV4-34 usage in COVID-19 patients as compared with HD that was more pronounced in active disease (Figure 6A). A comparable frequency pattern was found for isotype-switched IGHV4-34-AVY B cells in the single-cell dataset (Figure 6B). These switch events extended to different IGHV4-34 rearrangements (Figure 6C). Contrary to our initial hypothesis, we did not observe an enrichment of these rearrangements in the CD20^dim/−^ PB populations but in CD27^+^ memory-like populations independent of disease state (A6, corresponding to memory population R4 in recovered individuals) (Figure 6D). Because memory B cells can (re-)differentiate into short-lived antibody-secreting cells that produce autoantibodies or modulate autoimmunity (Hofmann et al., 2018; Jourdan et al., 2009), we searched for transcriptomic signatures related to these processes. Gene expression in the memory-like population with the highest proportion of switched IGHV4-34-AVY sequences (A6) was characterized by upregulation of IFN-responsive genes *IFIT2* and *ISG15* as well as *CD80*, *CD82*, *CR1* (*CD35*), *CD11b*, and *EBI3* (Figure 6E). In addition, we noticed enrichment of switched IGHV4-34-AVY sequences in another memory subpopulation, namely atypical memory B cells (aTMs). This memory subset is known to be associated with chronic infection and autoimmunity (Knox et al., 2019) and was found in the *ZBTB32*^+^/*CD27*^low^ memory compartment of population A1 (Figure 3C). It expressed *CD11c* (*ITGAX*) and *SOX5* but—aberrantly—showed low *T-bet* (*TBX21*) expression (Figure 6F). Interestingly, this aTM population was lost in patients after recovery from COVID-19 and appeared to be replaced by a new aTM population with *T-bet* expression as part of the memory population R3 (Figure 6F).

## Discussion

Perfect world humoral responses to vaccines or natural infection generate pathogen-specific long-lived plasma cells that produce high-affinity antibodies that protect the individual from reinfection over a lifespan. Yet, many pathogens induce inefficient B cell responses that do not lead to lasting immunity or otherwise require repetitive infection for their generation. Moreover, infections and exposure to opportunistic organisms have been recognized as a trigger for the initiation of autoimmunity or autoimmune flares (Chakravarty, 2008; Wucherpfennig, 2001). Currently, the molecular and cellular underpinnings of such inefficient or harmful B cell responses are not fully understood.

Here, we used COVID-19 as disease model to study B cell responses and their consequences for the generation of immunological memory and immunopathology. We chose COVID-19 for several reasons: first, the emergence of the SARS-CoV-2 virus in late 2019 excluded prior exposure (and consequently prior selected memory) to this virus in our patients. Second, early data on the SARS-CoV-2-induced B cell response suggested some features of unclear biological significance such as high peripheral PB counts (Bernardes et al., 2020; De Biasi et al., 2020; Kuri-Cervantes et al., 2020; Mathew et al., 2020) and avoidance of GC reactions (Kaneko et al., 2020) with only low levels of SHM in SARS-CoV-2 antibodies (Galson et al., 2020; Kaneko et al., 2020; Kreer et al., 2020; Schultheiss et al., 2020; Seydoux et al., 2020; Woodruff et al., 2020).

As a central technique, we performed combined single-cell RNA and V(D)J sequencing and found considerable expansions of oligoclonal PBs. Reflecting the ontogenetic dead-end that differentiated, mostly short-lived PBs represent, their transcriptional program was characterized by the loss of factors mediating B cell activation and differentiation as well as cell proliferation while biosynthetic programs needed for extensive antibody production were upregulated. The PB populations expressed the Pax5-repressed gene *TMSB10* (Liu et al., 2020; Pridans et al., 2008) and displayed signatures of an ongoing IFN response mirrored by high expression of *IFITM1* (Yang et al., 2007), *ISG15* (Perng and Lenschow, 2018), *IFNG-AS1* (Petermann et al., 2019; Rankin et al., 2020; Stein et al., 2019), and *NKBIZ* (Ishiguro-Oonuma et al., 2015). ISG15-secreting PBs have been described as a proinflammatory feature of active SLE (Care et al., 2016). Because ISG15 acts as chemoattractant for neutrophils (Perng and Lenschow, 2018), their increased infiltration at the site of infection (Chen et al., 2020; Reusch et al., 2021) might be fueled by the extensive PB response. Together with their expression of the lymphocyte homing receptor CD62L—characteristic for PBs whose first antigen encounter occurred in the upper respiratory tract (Palkola et al., 2016; Seong et al., 2017)—our findings suggest that PBs are direct actors in SARS-CoV-2 pathophysiology.

Despite a significant correlation of autoantibodies and PB counts, our analysis suggested that some memory subsets, but not PBs, are enriched in potentially autoreactive B cells. In the B cell subset containing most autoreactive BCR sequences (memory-like subset A6), autoimmune features were also noted on the transcriptional level including upregulation of *ISG15* and *CD80*. ISG15 expression is an autoimmune feature of memory-like B cells from SLE patients (Care et al., 2016), and CD80^+^ B cells can rapidly differentiate into antibody-secreting cells upon rechallenge without generating GC reactions (Zuccarino-Catania et al., 2014). In addition, we noticed that the memory population A1 contained high amounts of atypical memory B cells (aTMs) with low *T-bet* (*TBX21*) expression in line with previous flow cytometry data from COVID-19 and malaria patients (Wildner et al., 2021). ATMs have been described as a functional divergent antigen-experienced subset that has been associated with age, chronic infection, and that may contribute to autoimmunity (Hao et al., 2011; Knox et al., 2019; Rakhmanov et al., 2014; Rubtsov et al., 2011). However, these proinflammatory effector functions mainly rely on the activity of T-bet. As also hypothesized by (Wildner et al., 2021), the finding of an expanded aTM population with low T-bet expression in active COVID-19 that vanishes after recovery argues for a dysfunctional phenotype that might deregulate protective T cell responses.

Both the expanded PB population and the memory pool appeared to emerge from the same naive IL-4 receptor and BAFF receptor positive B cell subset. In conjunction with our cytokine data showing high IL-4 and BAFF plasma levels especially in the PB^high^ subset of patients, it appears very likely that the B cell trajectories described here are driven by these cytokines. It has been previously recognized that IL-4 promotes B cell maturation and activation by rendering the target cell refractory to B-cell-receptor-mediated cell death and that BAFF breaks B cell tolerance (Avery et al., 2003; Robinson et al., 2019). In addition, IL-6 is thought to promote differentiation of antigen-specific B cells to PBs but not to fully differentiated plasma cells (Gonzalez-Garcia et al., 2006; Jego et al., 1999). BAFF, IL-4, and IL-6 are produced predominantly by innate immune cells (IL-6 also by fibroblasts and endothelial cells [Mauer et al., 2015]), and therefore, the B cell dysregulation observed in this disease may be driven by the innate response.

The potential clinical consequences of cytokine-triggered autoreactive B cell populations and plasma autoantibodies in COVID-19, however, remain unclear at this point. There are various mechanisms by which viral infections can induce autoimmune responses, including molecular mimicry, epitope spreading, or apoptosis of virus-infected cells liberating autoantigens (Smatti et al., 2019). Such often low-titer transient responses only rarely progress to an established autoimmune disease. After COVID-19, a variety of inflammatory and autoimmune diseases such as multisystem inflammatory syndrome in children (MIS-C), Kawasaki-like disease, toxic shock syndrome, and macrophage activation syndrome may occur (Galeotti and Bayry, 2020). Moreover, many adult patients develop long-term effects of COVID-19 such as fatigue, pain, and concentration deficit often as a post-acute syndrome termed long-COVID. The exact pathogenesis of all of these conditions is still awaiting further molecular definition. However, of the seven individuals with a follow-up of 12 weeks after symptom onset, long-COVID was restricted to the three individuals who showed plasma autoantibodies. COVID-19 disease severity was moderate in these seven patients, with all of them requiring hospitalization but no ICU treatment. The limited size of this patient subset does not allow firm conclusions about a potential pathophysiological role of autoantibodies in long-COVID, but future studies are warranted to further explore this potential link.

Together, our data indicate that some of the elevated cytokines in COVID-19 patients may induce B cell trajectories, leading to highly expanded PBs and dysfunctional memory populations with autoreactive properties that may be the cellular source of autoantibodies in COVID-19. The strong induction of PBs at the expense of an efficient GC reaction in the generation of SARS-CoV-2 epitope-specific B cells (Galson et al., 2020; Kaneko et al., 2020; Kreer et al., 2020; Schultheiss et al., 2020; Seydoux et al., 2020; Woodruff et al., 2020) may be one of the determinants of the relatively short half-life of protective antibodies in this disease.

### Limitations of the study

One of the limitations of this study is the relatively small number of patient samples that was investigated by single-cell sequencing and especially the lack of follow-up clinical data for these cases that prevents a definitive appraisal of the potential clinical consequences of B cell dysregulation in COVID-19 and its postacute sequelae.

## STAR★Methods

### Key resources table

**Table:** 

REAGENT or RESOURCE	SOURCE	IDENTIFIER
**Antibodies**		

smCD3-APC-H7 (clone SK7)	Becton Dickinson (BD)	Cat# 560176; RRID:AB_1645475
CD19-PC7 (clone J3-119)	Becton Dickinson (BD)	Cat# IM3628
CD20-V450 (clone 2H7)	BioLegend	Cat# 302320; RRID:AB_10638575
CD27-BV605 (clone L128)	Becton Dickinson (BD)	Cat# 562656
CD38-APC-R700 (clone HIT2)	Becton Dickinson (BD)	Cat# 564980

**Biological samples**		

Peripheral blood of patients with active COVID-19 disease and after recovery	This paper	N/A
Peripheral blood of healthy individuals before and after vaccination with the seasonal flu vaccine (VaxxigripTetra2020/2021)	This paper	N/A

**Chemicals, peptides, and recombinant proteins**		

Phusion HS II	Thermo Fisher	Cat# 564980

**Critical commercial assays**		

LEGENDplex Human B Cell Panel (13-plex)	BioLegend	Cat# 740527
Anti-SARS-CoV-2-ELISA IgA	Euroimmun AG	Cat# EI 2606-9601 A
Anti-SARS-CoV-2-ELISA IgG	Euroimmun AG	Cat# EI 2606-9601 G
Anti-SARS-CoV-2-ELISA NCP	Euroimmun AG	Cat# EI 2606-9601-2 G
Chromium Next GEM Single Cell 5' Library and Gel Bead Kit v1.1	10X Genomics	Cat# 1000167
Tube, Dynabeads™ MyOne™ SILANE	10X Genomics	Cat# 2000048
Chromium Next GEM Chip G Single Cell Kit	10X Genomics	Cat# 1000127
Chromium Single Cell V(D)J Enrichment Kit, Human B Cell	10X Genomics	Cat# 1000016
Chromium Single Cell 5' Library Construction Kit,	10X Genomics	Cat# 1000020
Single Index Kit T Set A	10X Genomics	Cat# 1000213
ANAScreen	Orgentec	Cat# ORG 538
Rheumatoid Factor Screen	Orgentec	Cat# ORG 522S
Anti-Phospholipid Screen IgG/IgM	Orgentec	Cat# ORG 529

**Deposited data**		

NGS single-cell RNA sequencing data	This paper	EMBL-EBI Array Express: E-MTAB-11011
IGH NGS Sequencing data from COVID-19 patients	iReceptor Public Archive	ID IR-Binder-000001
NGS Sequencing data of SARS-CoV-2-specific BCRs	Ehling et al. (2021)	
NGS Sequencing data of SARS-CoV-2-specific BCRs	Galson et al. (2020)	
NGS Sequencing data of SARS-CoV-2-specific BCRs	Vita et al. (2019)	
NGS Sequencing data of human B cells	Dunn-Walters Lab (Stewart et al., 2021)	EMBL-EBI: E-MTAB-9544

**Oligonucleotides**		

BIOMED2-FR1 (IGH)	van Dongen et al. (2003)	N/A

**Software and algorithms**		

R Studio version 3.5.1	RStudio, Boston, USA	https://rstudio.com/products/rstudio/
GraphPad Prism 8.0.2	GraphPad Software, La Jolla, CA, USA	https://www.graphpad.com/scientificsoftware/prism/
FlowJo v10	Becton Dickinson	https://www.flowjo.com/solutions/flowjo
MiXCR (3.0.8)	Bolotin et al. (2015)	https://mixcr.readthedocs.io/en/master/
Cell Ranger software (v3.1.0)	10X Genomics	https://support.10xgenomics.com/single-cell-gene-expression/software/downloads/latest
Seurat (v 3.2.0)	Satija Lab	https://satijalab.org/seurat/
Slingshot 1.4.0	Street et al. (2018)	https://bioconductor.org/packages/release/bioc/html/slingshot.html
IMGT/HighV-QUEST	Brochet et al., 2008	http://www.imgt.org/HighV-QUEST/home.action
FastTreeMP	Price et al., 2009	http://www.microbesonline.org/fasttree/
Archaeopteryx viewer (0.9928 beta)	Han and Zmasek, 2009	https://sites.google.com/site/cmzmasek/home/software/archaeopteryx

### Resource availability

#### Lead contact

Further information and requests for resources and reagents should be directed to and will be fulfilled by the Lead Contact, Mascha Binder (mascha.binder@uk-halle.de).

#### Materials availability

This study did not generate new unique reagents.

### Experimental model and subject details

#### Patients and samples

Blood samples were drawn from 23 symptomatic SARS-CoV-2 PCR-positive Inpatients at the University Medicine Halle (Saale) between March and December 2020 and 10 PCR-negative recovered individuals (Table S1). Thirteen of these patients required intensive care. Up to 17 follow-up blood samples were available, 65 from the active disease phase, 16 from the convalescent phase. As control, five patients with bacterial pneumonia and 10 healthy individuals vaccinated with the seasonal influenza vaccine (VaxxigripTetra2020/2021) were included. The studies were approved by the institutional review board (approval numbers 2020-039 and 11/17) and were conducted in accordance with the ethical principles stated by the Declaration of Helsinki. Informed written consent was obtained from all participating patients or legal representatives.

### Method details

#### Flow cytometry

Peripheral blood mononuclear cells (PBMCs) were isolated from peripheral blood by density gradient centrifugation with Biocoll cell separation solution (Biochrom AG, Berlin, Germany) and frozen in FCS + 10% DMSO. For analysis, PBMCs were thawed and washed twice with PBS. One million cells were stained with smCD3-APC-H7 (clone SK7, BD), CD19-PC7 (clone J3-119, Beckman Coulter), CD20-V450 (clone 2H7, BioLegend), CD27-BV605 (clone L128, BD) and CD38-APC-R700 (clone HIT2, BD for 30 min at room temperature. Flow cytometry was performed on a BD FACSLyric^TM^ and data was analyzed using FlowJo software (version 10).

#### Quantification of plasma antibody titers using ELISA

Plasma levels of antibodies directed against the S1 domain of the spike (S) protein and the nucleocapsid protein (NCP) of SARS-CoV-2 were determined using the Anti-SARS-CoV-2-ELISA IgA/IgG and Anti-SARS-CoV-2-NCP-ELISA kits from Euroimmun AG (Lübeck, Germany) according to the manufacturer's instructions. Quantification of rheumatoid factor (RF), antinuclear antibodies (ANA) and phospholipid antibodies (aPL) was performed using the Rheumatoid Factor Screen (detects IgG, IgA and IgM RFs), ANAscreen (detects SS-A 60, SS-A 52, SS-B, RNP-70, Sm, RNP/Sm, Scl-70, centromere B and Jo-1 IgGs) and Anti-Phospholipid Screen IgG/IgM (detects cardiolipin, phosphatidylserine, phosphatidylinositol, phosphoglycerides und β2-glycoprotein 1 IgGs/IgMs) kits from Orgentec (Mainz, Germany). Readouts were performed at 450 nm using a Tecan Spectrophotometer SpectraFluor Plus (Tecan Group Ltd., Männedorf, Switzerland).

#### Cytokine responses

Plasma was isolated by centrifugation of whole blood for 15 min at 2,000 x g, followed by centrifugation of 12,000 x g for 10 min. Samples were stored at - 80°C before use. Cytokine plasma levels were detected using the bead-based LEGENDplex Human B Cell Panel (13-plex) immunoassay from BioLegend (via Biozol, Munich, Germany) according to the supplier's suggestions. Data was acquired using the BD FACSCelesta flow cytometer and analyzed with the BioLegend LEGENDplex software.

#### Isolation of CD19+ B cells for single-cell RNA-seq

The seven patients that underwent single-cell analyzes are marked with an asterisk in Table S1. Untouched B cells were isolated from PBMCs via magnetic separation using the B Cell Isolation Kit II (Miltenyi). Viability ranged between 92-98% as determined with the Cell Viability Analyzer Vi-Cell XR (Beckman Coulter). After *in vitro* staining with APC anti-CD19 (BD) and amcyan live/dead stain, viable CD19+ cells were sorted with a 70 μm nozzle into PBS plus 0.1% BSA at a concentration of 100 cells/μl. Cells were processed within 1 h after collection according to 5′ GEX and V(D)J protocols suggested by 10x.

#### Pre-processing of single-cell RNA-seq data

The Cell Ranger software pipeline (v5.0.1, 10x Genomics) was used for demultiplexing and read count annotation to the human reference genome (refdata-cellranger-GRCh38-3.0.0, 2020-A (July 7, 2020)). The resulting filtered feature-barcode matrix, which contains all gene expression counts per cellular barcode, was further processed in R using the Seurat (v 3.2.0) package(Stuart et al., 2019). To remove low quality cells and doublets, all cells in which less than 200 or more than 2,500 genes were detected and/or cells where mitochondrial genes accounted for 10 or more percent of all detected genes were filtered out. The remaining cells were log-normalized and globally scaled to factor 10,000. We calculated features that exhibited high variation between cells with the function FindVariableFeatures and method “vst”. Prior to dimension reduction, we used ScaleData as linear transformation. With a principal components analysis (PCA) and elbow plot over the variable features we estimated the dimensionality of the dataset. The majority of robust signals was captured in the first 10 principal components. To cluster the cells we used function FindNeighbors to construct a KNN graph based on Euclidean distance using the dimensions previously defined. Using FindClusters cells were grouped together to graph based clusters. For visualization we used UMAP as dimension reduction plot.

#### Combining gene expression datasets

In order to integrate the single patient datasets we used function *merge* from R package Seurat. After finding integration anchors by use of function *FindIntegrationAnchors* a log normalized integrated dataset was created applying function *IntegrateData*. Data was scaled and a PCA was calculated on variable features of each object. Clusters were defined using function *FindNeighbors* and *FindClusters*. UMAP plot was generated using function *RunUMAP* and *DimPlot*. Features were visualized on UMAP plots with function *FeaturePlot* and differential expression in PB cluster was calculated using *FindMarkers*. We generated volcano plots with package EnhancedVolcano. Gene set enrichment analysis was performed using the Gene Ontology (GO) Resource at http://geneontology.org/ with the top 200 enriched genes per cluster.

#### BCR-seq data processing and integration

BCR-seq data for each sample were assembled using the Cell Ranger software (v3.1.0, 10xGenomics) with the command *cellranger vdj* and the reference genome (refdata-cellranger-vdj-GRCh38-alts-ensembl-3.1.0). For each sample, Cell Ranger generated an output file (filtered_contig_annotations.csv) containing BCR heavy and light chain CDR3 nucleotide sequences for individual cells identified by their barcodes. The R package scRepertoire (v1.2.1) (Borcherding et al., 2020) was used to further combine the contig_annotation data of different samples to a single list object (function *combineBCR*). The combined BCR contig list file was then integrated with the corresponding Seurat object of the scRNA-seq data using the function *combineExpression* (cloneCall="gene"). Only cells with BCR and scRNA-seq data were kept for downstream clonotype analysis. The clonotype was defined according to the genes comprising the BCR and the nucleotide sequence of the CDR3 region.

#### Mining for SARS-CoV-2-specific B cell receptor sequences

We searched all V(D)J rearrangements from our single-cell dataset for 258 neutralizing and 478 non-neutralizing SARS-CoV-2 rearrangements retrieved from CoVAbDab(Galson et al., 2020) on December 7^th^ 2020 and from (Ehling et al., 2021) and also mined the IEDB database(Vita et al., 2019). Sequences with identical or highly similar CDR3 amino acid sequence (Levenshtein distance of ≤2) were counted as hits.

#### Evolutionary analysis of IGHV4-34-AVY B cells from single-cell dataset

Repertoire-wide evolutionary analysis of B cells was performed using approximately maximum-likelihood trees (VanDuijn et al., 2017). The amino acid sequence of each clone was gapped according to the IMGT unique numbering using HighV-QUEST (Brochet et al., 2008). Phylogenetic trees were inferred with FastTreeMP (Price et al., 2009) and visualized with Archaeopteryx (v 0.9928).

#### Pseudotime analysis of B cell trajectories

Trajectories were constructed using the R package slingshot (v1.4.0)(Street et al., 2018) as described in (Fletcher et al., 2017). Slingshot was run on normalized log-count data and UMAP dimensions. Pseudotime trajectories were plotted over UMAP with function *slingCurves*. Differential expression between clusters along each trajectory was carried out using *FindMarkers* from package Seurat applying the one-versus-all comparison over scaled data. Average expression of resulting gene sets was illustrated with pheatmap package.

#### Bulk NGS immunosequencing and data analysis

To determine the entirety of the clonal V(D)J rearrangements of peripheral B cell receptors (B cell repertoire), all acquired blood samples underwent next-generation sequencing of the IGH genetic locus. In brief, genetic loci were amplified together in a multiplex PCR using BIOMED2-FR1 (IGH) primer pool and 250–500 ng of genomic DNA (van Dongen et al., 2003). The primers were purchased from Metabion International AG (Martinsried, Germany). Two consecutive PCR reactions were performed to generate fragments tagged with Illumina-compatible adapters for hybridization to the flow cell and 7 nucleotide barcodes for sample identification. All PCRs were performed using Phusion HS II (Thermo Fisher Scientific Inc., Darmstadt, Germany). After gelelectrophoretic separation, amplicons were purified using the NucleoSpin® Gel and PCR Clean-up kit (Macherey-Nagel, Düren, Germany), quantified on the Qubit platform (QIAGEN, Hilden, Germany) and pooled to a final concentration of 4 nM. The quality of the amplicon pools was controlled on an Agilent 2100 Bioanalyzer (Agilent Technologies, Böblingen, Germany) before undergoing NGS. Annotation of IGH loci rearrangements was computed with the MiXCR framework (3.0.8)(Bolotin et al., 2015). As reference for sequence alignment the default IMGT library v3 was used. Non-productive reads and sequences with less than 2 read counts were not considered for further analysis. Each unique complementarity-determining region 3 (CDR3) nucleotide sequence was defined as one clone. All repertoires were normalized by proportionally downsizing read counts to 20,000 productive reads using a scaling factor x to preserve repertoire structures for inter-individual comparison. The scaling factor x for each repertoire was calculated as 20,000/total read count of the individual repertoire. The read counts for each clonotype within a given repertoire were then multiplied by x. Only clonotypes with two or more reads were used for the normalized repertoire. To assess the physiochemical properties of recombined IGH sequences, we calculated the grand average of hydropathy index (GRAVY)(Kyte and Doolittle, 1982) using Brepertoire(Margreitter et al., 2018). The GRAVY indices for each cohort were plotted against the cumulative relative frequency of each CDR3 amino acid sequence (1/total number of clones within cohort). All analyses and data plotting was performed using R version 3.5.1.

### Quantification and statistical analysis

Differences in plasma cytokine levels and antibody titers were studied by ordinary one-way ANOVA followed by post-hoc testing (Tukey’s multiple comparisons test) and two-tailed student’s t-test using GraphPad Prism 8.0.2 (GraphPad Software, La Jolla, CA, USA). Ranges of p values are indicated with asterisks: ∗p<0.05; ∗∗p<0.01; ∗∗∗p<0.001; ∗∗∗∗p<0.0001.

## Data Availability

Sequencing data have been deposited in the ArrayExpress database at EMBL-EBI (www.ebi.ac.uk/arrayexpress) under accession number E-MTAB-11011.
