# Supplementary material for: Maturation trajectories and transcriptional landscape of plasmablasts and autoreactive B cells in COVID-19
Source: iScience. 2021 Oct 23;24(11):103325. doi: 10.1016/j.isci.2021.103325 (PMC8536484; doi:10.1016/j.isci.2021.103325)
Supplement: Document S1. Figures S1–S5 and Table S1 [file mmc1.pdf]

iScience, Volume *24*

Supplemental information

Maturation trajectories and transcriptional landscape of plasmablasts and autoreactive B cells in COVID-19

Christoph Schultheiß, Lisa Paschold, Edith Willscher, Donjete Simnica, Anna Wöstemeier, Franziska Muscate, Maxi Wass, Stephan Eisenmann, Jochen Dutzmann, Gernot Keyßer, Nicola Gagliani, and Mascha Binder

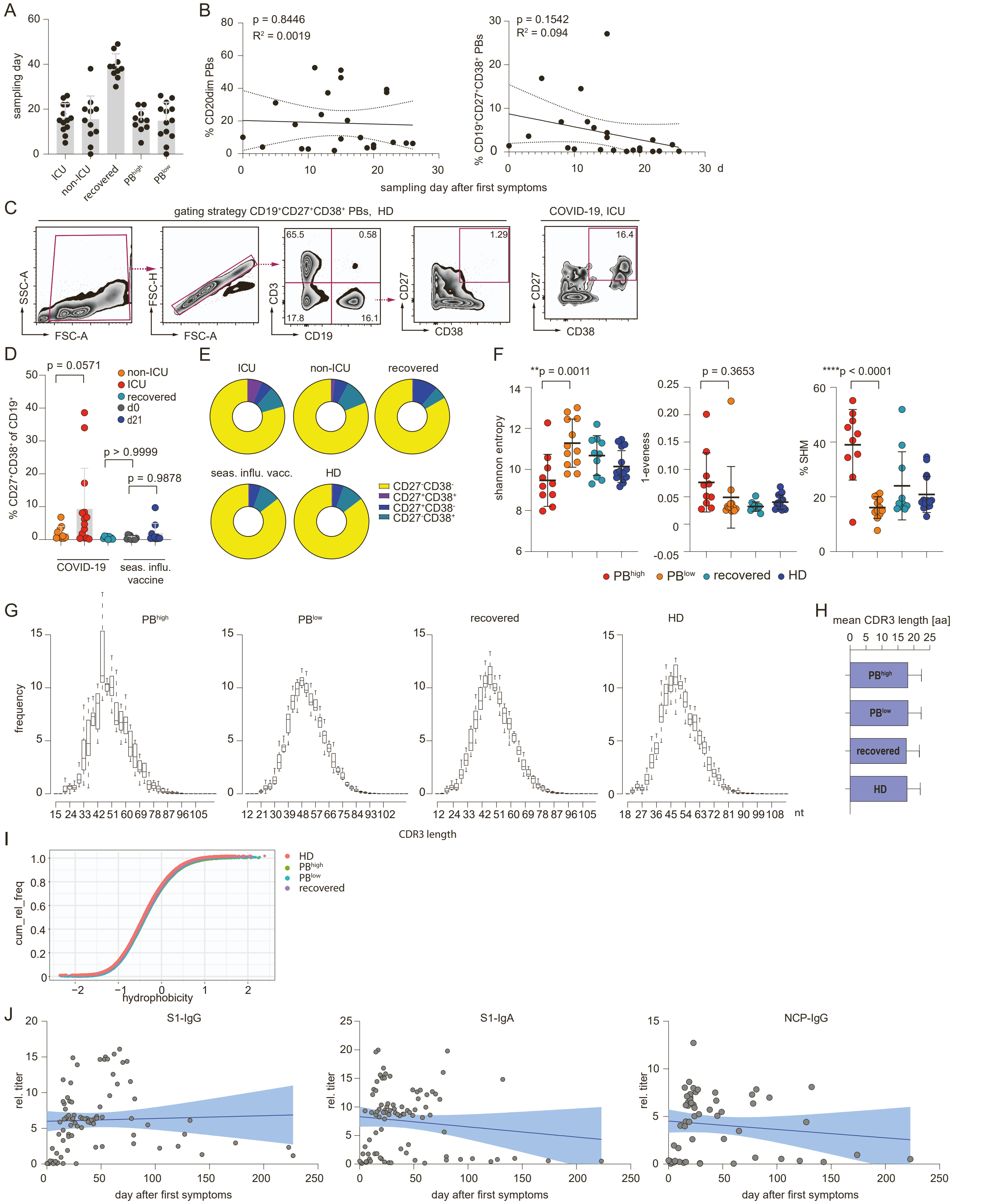

Figure S1. Identification and immunogenetic characterization of plasmablast expansions and decay of SARS-CoV-2 directed antibody titers, related to Figure 1.

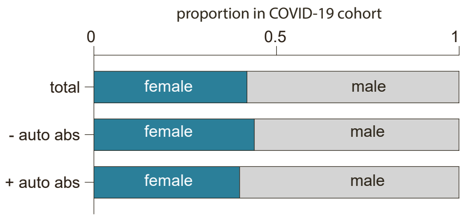

Figure S2. Gender proportion in COVID-19 patients with autoantibodies, related to Figure 2.

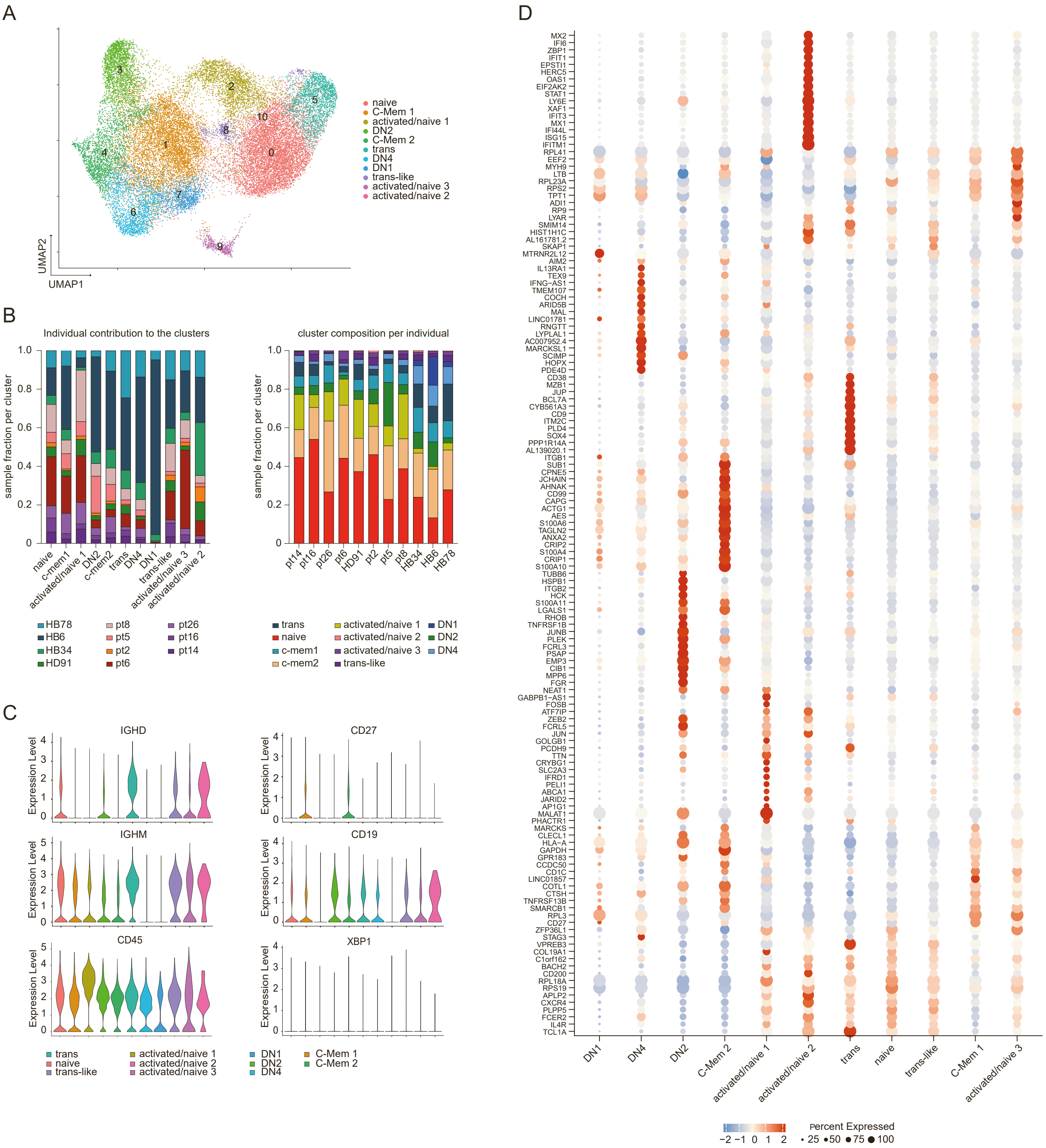

Figure S3. Analysis of integrated CD19⁺ B cell data sets including B cells from COVID-19 patients, related to Figure 3.

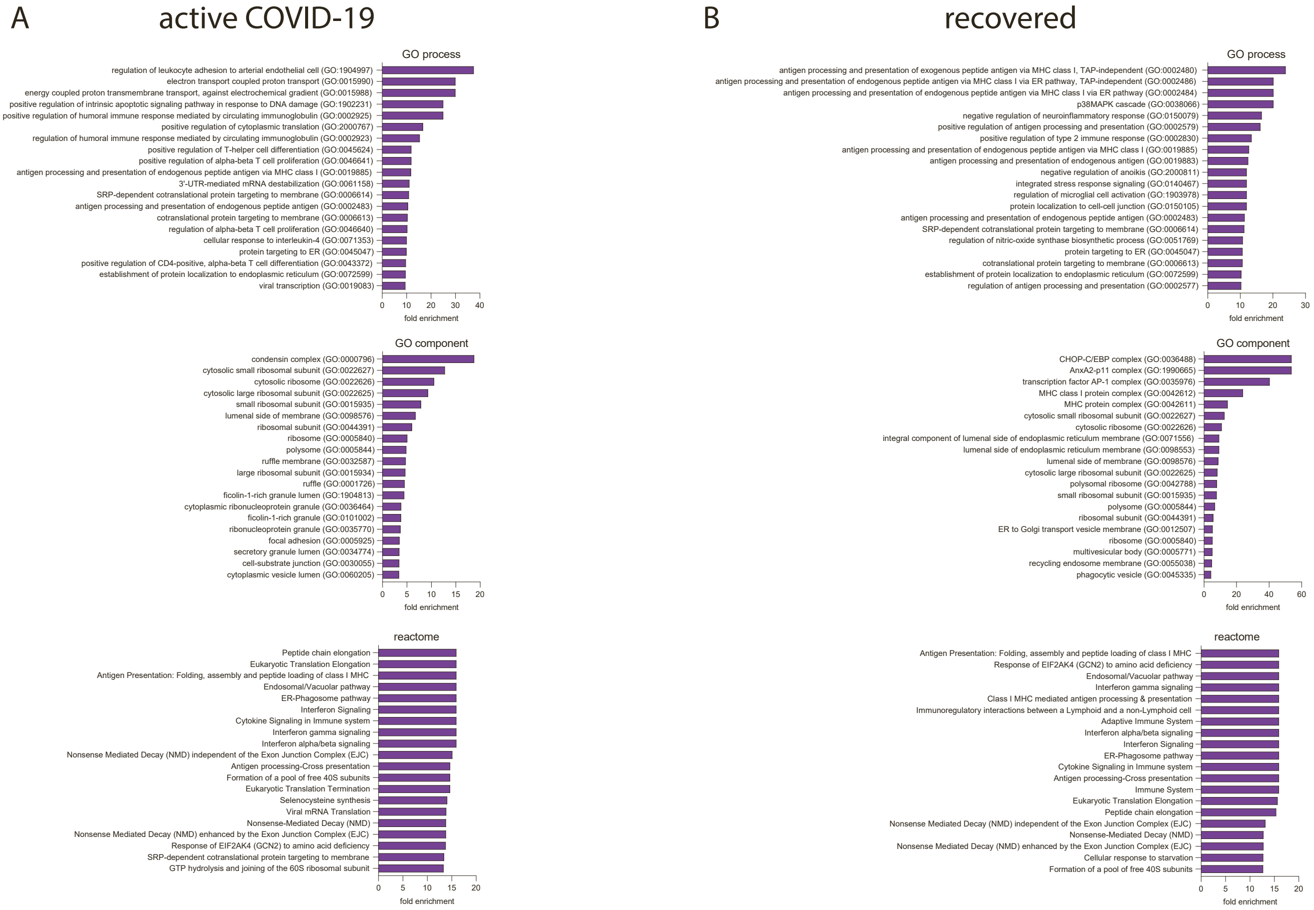

Figure S4. Pathway enrichment analysis of CD19^{+} B cells from active COVID-19 patients and after recovery, related to Figure 3.

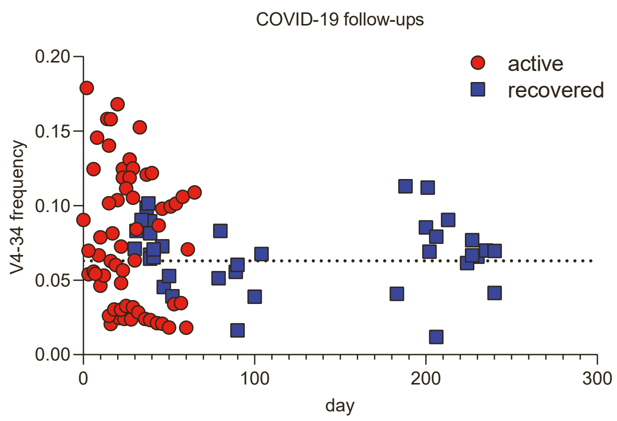

Figure S5. IGHV4-34 gene usage in long-term follow-up samples, related to Figure 6.

Table S1. Patient characteristics, related to Figures 1-2 and STAR Methods.

sample	sex	age	sampling	intervention	hospitalization	fatal	comorbidities
pt1	m	66	d14	ECMO	ICU	yes	AML; prostate carcinoma;
pt2*	m	61	d11	ECMO	ICU	yes	AML; aHT; CKD; cardiomyopathy
pt3*	m	76	d5	vent	ICU	yes	T-NHL; AIHA; hypothyreodism; RA
pt5*	m	67	d26	ECMO	ICU	no	diabetes; aHT; hypothyreodism
pt6	f	68	d23	SB	non-ICU	no	aHT; hypothyreodism; adrenocortical adenoma
pt7	f	70	d20	SB	non-ICU	no	diabetes; aHT; steatosis; cholelithiasis
pt8*	m	41	d18	ECMO	ICU	yes	aHT; Factor XIII deficiency
pt9	m	68	d22	ECMO	ICU	yes	hypothyreodism; eHT
pt10	m	66	d25	ECMO	ICU	yes	Rhabdomyolysis; AKI; COPD; Splenomegaly
pt24	f	85	d20	SB	non-ICU	yes	aHT, diabetes; Campylobacteriosis
pt25	m	65	d20	ECMO	ICU	yes	Myasthenia gravis
pt44	m	74	d16	ECMO	ICU	yes	
pt65	f	76	d15	SB	non-ICU	no	aHT; CKD; Reynaud synd.
pt67	f	23	d15	SB	non-ICU	no	
pt68	f	23	d3	SB	non-ICU	no	
pt69	f	62	d10	SB	non-ICU	no	Renal cell carcinoma; aHT
pt70	m	68	d0	SB	non-ICU	no	aHT; CAD; MI; diabetes; stroke
pt100	m	74	d19	ECMO	ICU	yes	PE
pt106	m	80	d22	ECMO	ICU	yes	diabetes; PAD; COPD
pt116	f	75	d8	SB	non-ICU	no	
pt117	f	80	d13	ECMO	ICU	yes	aHT; obesity
pt118	m	57	d15	vent	ICU	no	aHT; diabetes; PAD
pt125	f	51	d12	SB	non-ICU	no	
recovered							
pt13	f	46	d36	SB	non-ICU	no	RA
pt14*	m	50	d38	SB	non-ICU	no	
pt16*	m	37	d37	SB	non-ICU	no	aHT
pt26*	m	49	d49	SB	non-ICU	no	asthma
pt32	f	28	d34	SB	non-ICU	no	
pt33	m	34	d39	SB	non-ICU	no	
pt34	m	40	d39	SB	non-ICU	no	
pt35	f	62	d47	SB	non-ICU	no	
pt55	f	38	d41	SB	non-ICU	no	
pt57	m	60	d30	SB	non-ICU	no	

d = sampling day after first symptoms, SB = spontaneous breathing, ICU = intensive care unit, ECMO = extracorporeal membrane oxygenation, vent = mechanical ventilation, aHT = arterial hypertension, eHT = essential hypertension, AML = acute myeloid leukemia, AKI = acute kidney injury, AIHA = autoimmune hemolytic anemia, CAD = coronary artery disease, CKD = chronic kidney disease, COPD = chronic obstructive pulmonary disease, MI = myocardial infarction, PE = pulmonary embolism, PAD = peripheral artery disease, RA = rheumatoid arthritis, T-NHL = T Non-Hodgkin lymphoma, * = included in single-cell RNA sequencing.
